# Lipid-rafts remain stable even after ionizing radiation induced disintegration of β1 integrin containing focal adhesions

**DOI:** 10.1186/s13104-017-3032-8

**Published:** 2017-12-06

**Authors:** Laura Babel, Larissa Kruse, Steven Bump, Markus Langhans, Tobias Meckel

**Affiliations:** 1 Membrane Dynamics, Department of Biology, Technische Universität Darmstadt, Schnittspahnstrasse 3, 64287 Darmstadt, Germany; 2 GRK 1657, Molecular and Cellular Responses to Ionizing Radiation, Technische Universität Darmstadt, Darmstadt, Germany

**Keywords:** Lipid raft, Membrane dynamics, Integrin, 3D cell culture, Single molecule microscopy

## Abstract

**Objective:**

Adhesion of cells to the extracellular matrix is facilitated by integrin receptors. We recently found that a nanoscale organization of plasma membrane located integrins containing the β1 subunit is responsible for an enhanced radio-resistance in 3D cultured cells over cells grown in 2D. While ionizing radiation is known to have broad effects on the lipid composition of the plasma membrane and their organization in lipid-rafts, it is not clear whether the effects of ionizing radiation on the nanoscale clustering of integrins is lipid-raft dependent.

**Results:**

Using single molecule microscopy we can show that β1 integrins colocalize with cholesterol in lipid-rafts. Ionizing radiation, as an extrinsic stressor, causes the separation of β1 integrins from cholesterol lipid raft suggesting that the effects of ionizing radiation on the clustering of β1 integrins are lipid-raft independent.

**Electronic supplementary material:**

The online version of this article (10.1186/s13104-017-3032-8) contains supplementary material, which is available to authorized users.

## Introduction

It has been reported that cells embedded in a 3D matrix are more radio-resistant than those cultured in a standard, monolayer 2D cell culture. This phenomenon of increased radioresistance in a 3D matrix has been termed cell-adhesion-mediated-radio-resistance (CAM-RR) [1–3]. We recently found that clustering of integrin β1 is a sensitive and robust indicator of radio-resistance [5]. Cells cultured under standard (2D) conditions are not able to organize integrin receptors, which facilitate cell adhesion [4], into firm and stable clusters. They display a rather loose and dynamic cluster organization of the ECM (extracellular matrix) receptor. On the contrary, cells embedded in an ECM, exhibit a stable integrin organization. Exposure of 2D cultured cells to ionizing radiation causes already at low doses a severe disturbance of the unstable integrin organization. The same treatment has no perceivable effect on the well clustered organization of integrins in 3D cultured cells. On the basis of these data we could therefore causally link the radioresistance of 3D cells to their ability to maintain stable clusters [5].

It is well accepted that IR has profound effects on the PM beyond integrin clustering. Mainly lipid peroxidation, generation of ceramides and its organization in ceramide lipid rafts are well studied. Ionizing irradiation generates reactive oxygens (ROS) which damage the integrity of the membrane and modify lipids directly with the consequence of profound effects on lipid signalling, organization and dynamics [6–8]. Physical differences in lipids such as chain length, chain geometry and head groups cause an in-homogeneous distribution of membrane components and an aggregation in defined domains. In particular sphingolipids and cholesterol aggregate in microdomains known as lipid rafts [9, 10]. Lipid rafts are highly dynamic structures, of 10–200 nm size, which limit the free diffusive properties of biomembranes as proposed by Singer and Nicolson in their fluid mosaic model [11]. These micro structures are known to function as parts of signaling cascades or as platforms for membrane protein clustering; in this way they modify protein activity [12]. Proteins localize in lipid rafts either because of direct interaction with the lipid head group or in response to physical forces such as lateral pressure, charge interactions or the local curvature of the membrane [13]. It is known that integrins and cholesterol rich regions colocalize [14, 15] suggesting that integrins are predominantly localized in lipid rafts.

Here we use ionizing radiation as a tool to disrupt integrin clustering and native co-cluster organization of integrin β1 with cholesterol. In the case that lipid rafts are responsible for the effects on integrin clustering, we expect that: (i) the before mentioned cholesterol raft organization is ECM dependent, and (ii) that IR breaks cholesterol raft organization in concert with integrin cluster break down.

To our surprise, we found that integrins disintegrate in a lipid raft independent manner. Even after high doses of IR cholesterol remained in clusters, while β1 integrins were separated from their raft localization.

## Main text

A detailed description of the methods, with references to [16–24], can be found in Additional file 1.

### Membrane mobility and lipid raft organization are strongly affected by the cell culture condition

To investigate the mobility and nanoscale organization of the PM of cells as a function of their culture conditions, we analyzed an isoprenyl anchored membrane protein (CAAX-mCherry) as a reporter for membrane fluidity [25] and clustering of cholesterol as a marker for lipid rafts in 2D and 3D cultured cells.

**Fig. 1:**
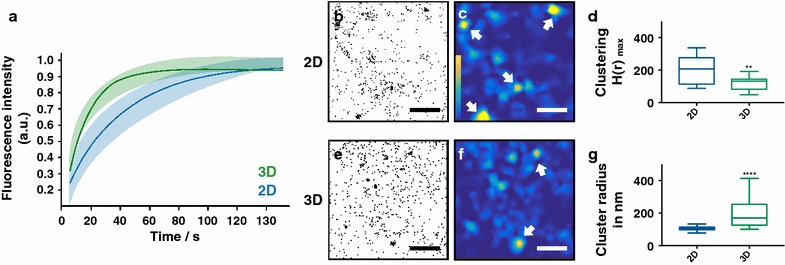
2D vs. 3D cell culture conditions have a strong impact on the membrane mobility and cholesterol raft organization. **a** FRAP curves of PM located CAAX-mCherry of 2D (blue, n = 8) and 3D (green, n = 9) cultured OV-MZ-6 cells. Exponential fits of recovery dynamics and the standard derivations. **b–g** Single molecule data of cholesterol stainings of 2D and 3D MEF cells as well as corresponding cluster analysis. **b**, **e** Scatter plots show all detected cholesterol molecules, **c**, **f** corresponding heat maps visualize clustered (yellow) and unclustered (dark blue) regions, arrows indicate cholesterol rafts. Scale bar is 1 μm. Statistical analysis with the Ripley’s K function reveals the clustering (**d**) and the cluster size (**g**). Statistical analysis was performed with a Mann–Whitney test. **p ≤ 0.01 and ****p ≤ 0.0001

For the analysis of membrane mobility, cells were transfected with CAAX-mCherry and the mobility of this protein was monitored by FRAP (fluorescence recovery after photobleaching). The recovery curves reveal (Fig. 1a) that 3D cultured cells possess a higher membrane fluidity; fluorescence recovery occurred faster than in 2D cultured cells. An exponential fit yields a halftime recovery value of 10.63 s and a mobile fraction of 88% for 3D cells. Corresponding analysis on the top membrane of 2D cultured cells reveal a similar value for the mobile fraction of 83% but a much longer halftime recovery (27.41 s). These results show that already the basic fluidity of the PM differs between 2D and 3D cultured cells. Since basically all signaling cascades relay on a dynamic (re)organisation of the PM [26], we can assume that the dynamics of PM located signaling are bound to differ in 2D and 3D cultured cells.

To further investigate if lipid rafts, often attributed as the organizers of PM located signaling activity [10], are affected by the different culture conditions, 2D and 3D cultured cells were stained with a cholesterol affine fluorescent probe (Dronpa-θD4). Cells were than imaged by single molecule localization microscopy and quantitatively assessed by a detailed cluster analysis (Fig. 1b–g). Because it was unfortunately not possible to completely immobilize lipids via chemical fixation [27, 28], we assured that the remaining mobility was not altering the cluster organization (Additional file 1: Figure S1). The effects of the two cell culture conditions on cholesterol raft organization can be directly recognized by a visual inspection of the single molecule localization results. Each point in the scatter plot of Fig. 1b, e represents an individual detection of a cholesterol molecule. Both scatter plots show that cholesterol is organized in micro-domains; this is evident from a higher density of the signals. These domains, long know as sphingolipid-cholesterol lipid rafts [29] vanish upon cholesterol depletion (Additional file 1: Figure S2). To quantify the visual impression we performed a Ripley’s K function cluster analysis. This function counts the number of signals that fall within a defined radius of each detected signal. By plotting this number versus the respective radii a distribution (H-plot) is yielded. The first local maximum in this plot represents the most prominent cluster formation of the data set. The height of this maximum provides: (i) a measure of the clustering (H(r) _max_) and (ii) the position the cluster radius (r _max_). For a better visualization of the single molecule localizations, 2D plots of the H(r) _max_ values are represented as heatmaps. They identify clustered regions with a higher density of signals as yellow areas (Fig. 1c, f). The heat maps reveal that 2D cultured cells possess more cholesterol rafts with a higher degree of clustering. The quantitative K function analysis support these findings (Fig. 1d, g). 2D cultured cells exhibit a significantly (**p ≤ 0.01) higher degree in clustering compared to 3D cultured cells. The former also have a smaller radius (****p ≤ 0.0001: 2D 〜 100 nm, 3D 〜 160 nm).

Taken together the data show that not only the membrane mobility but also the organization of lipids into rafts are remarkably affected by the cell culture condition. This suggests even more that PM located signaling activity differs in 2D and 3D cultured cells. The results of these experiments are well in line with our previous findings in that not only integrin β1 clustering, but also the number of the immediate downstream signaling partner pFAK (phosphorylated focal adhesion kinase) differs significantly between the cultured conditions. 2D cultured cells presumably possess an impaired signalling efficiency [5]. At this point we can conclude that the localization and organization of cholesterol rafts differ in cells depending on whether they were cultured in 2D or 3D.

### Lipid rafts—other than integrins—do not change their cluster organization in response to high dose irradiation

**Fig. 2:**
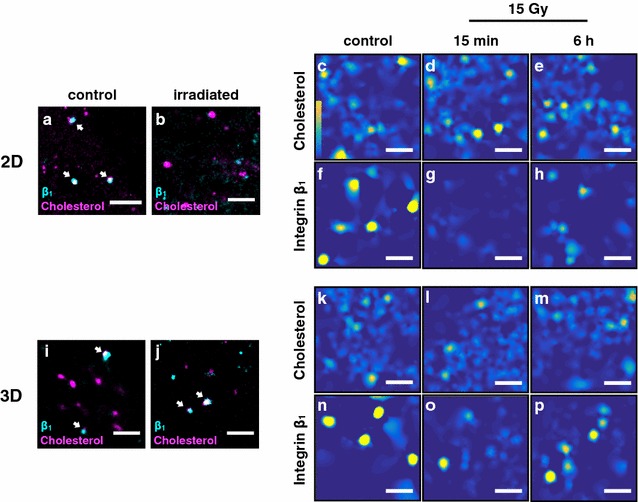
Effects of high dose irradiation on the integrin β1-cholesterol raft colocalization of 2D and 3D cultured MEF cells. **a**, **b**, **i**, **j** Superresolution images of PM located integrin β1 (cyan) and cholesterol (magenta) colocalizations of a 2D control cell (**a**), a 2D cell irradiated with 15 Gy (X-ray) (**b**), a 3D control cell (**i**) and a 3D cell irradiated with 15 Gy (**j**). Cells were fixed 15 min after irradiation. Scale bar is 2 µm. Arrows indicate regions with integrin β1-cholesterol colocalization (white). **c–h** Heat maps visualize clustered (yellow) and unclustered (dark blue) regions of 2D cells stained for cholesterol (**c**–**e**) and integrin β1 (**f**–**h)**. Shown are heat maps of controls (**c**, **f**), cells irradiated with 15 Gy and fixed after 15 min (**d**, **g**) and after 6 h (**e**–**h**). Scale bar is 1 µm. **k**–**p** Corresponding data for 3D cultured cells

To examine whether the colocalization of integrin β1 and cholesterol is maintained after high dose irradiation, we stained cells in order to monitor both micro organizations. After co-staining the target domains cells were irradiated and imaged, followed by single molecule localization analysis. The data reveal a culture condition independent coclustering of cholesterol rafts and integrin β1 clusters (Fig. 2a, i).

Previously we found that 2D cultured cells have a less well organized status of integrin β1. These unstable clusters were easily disturbed even by low doses (2 Gy) of radiation. In contrast, the same IR dose turned out to be completely ineffective in 3D cultured cells for affecting the well clustered organization of integrins. Also a high dose of irradiation (15 Gy) leads in 2D cultured cells to a complete break down of integrin clusters while it causes only a partial disintegration in 3D cultured cells [5].

If IR induced integrin cluster break-down would mainly be determined by lipid rafts one would expect that the same treatment causes a simultaneous disintegration of both domains. 2D cultured cells, which were fixed 15 min after an irradiation with 15 Gy, exhibited a loss of integrin clusters and a decreased amount of integrins. The cholesterol raft organization on the other hand remained unaffected by this treatment (Fig. 2b). The results of these experiments show that the integrin cluster break-down is unrelated to the integrity of lipid rafts. Heat maps support this finding (Fig. 2c–h). While the clustering of cholesterol remains unchanged, integrin clusters and signals are lost 15 min after irradiation; they only partly regenerated after 6 h.

**Fig. 3:**
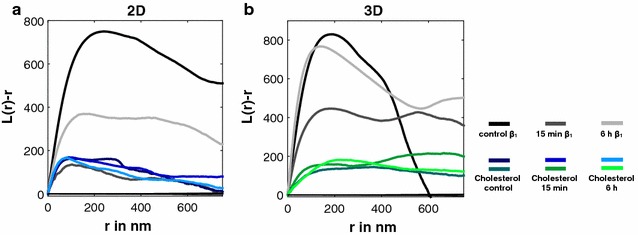
Effects of ionizing radiation on integrin β1 and cholesterol microdomain organization of 2D and 3D cultured MEF cells. H-Plots of datasets analyzed with Ripley’s K function for integrin β1 and cholesterol microdomains from 2D (**a**) and 3D (**b**) cultured cells. The peak heights (H(r) = L(r) − r) represent the degree of clustering (H(r) max) and their position the most frequent cluster size (r in nm). H-plots show results for controls and cells irradiated with 15 Gy fixed 15 min and 6 h after IR. Color code: integrin β1 control (black), integrin β1 15 min after IR (dark gray), integrin β1 6 h after IR (light gray), 2D cholesterol control (dark blue), 2D cholesterol 15 min after IR (mid-blue), 2D cholesterol 6 h after IR (light blue), 3D cholesterol control (dark green), 3D cholesterol 15 min after IR (mid-green) and 3D cholesterol 6 h after IR (light green). Also, an analysis of 100 random distributions of localizations containing the same number of signals as the control are plotted (confidence interval, gray)

In contrast to 2D cells, 3D cells not only maintain their clustered organization of β1 integrins after irradiation with high doses but also show a faster recovery. Irradiation with 15 Gy only triggers a slight decrease in integrin clustering and therefore also only a minor reduction of integrin-cholesterol coclustering (Fig. 2j–p) 15 min after IR. The effects are completely recovered after 6 h. As much as cholesterol rafts are not affected by high dose irradiation with 15 Gy in 2D cultured cells they also remain unaffected in 3D cultured cells. Following visual inspection of the images we used the Ripley’sK function to generate H-plots for quantification (Fig. 3). The H-plots reveal that the cholesterol organization is unaffected by high dose irradiation in a cell culture independent manner. Our detailed cluster analysis reveals that also parameters, such as cholesterol raft density and number of cholesterol microdomains do not change after irradiation (Additional file 1: Figure S3). These results demonstrate, that it is possible to separate a protein from its lipid raft localization by physical force like X-ray irradiation. This implies that independent forces underlie the co-organization of proteins and lipids in membrane clusters.

### Effects of IR on integrin β1 clustering are lipid raft independent

Taken together, we found that:

- Membrane dynamics and cholesterol raft organization differ between 2D and 3D cultured cells.
- The integrin-cholesterol raft colocalization is cell culture independent.
- Integrins can be separated from their lipid raft localization by an extracellular stressor.
- Cholesterol rafts remain surprisingly stable even after a sudden and complete disappearance of proteins, with which they colocalized before a treatment.

Even after exposing cells to high doses of IR, cholesterol remains clustered in the PM. In contrast, integrin clusters disintegrate in response to this treatment and loose their association to lipid rafts, often referred to as “organizing platforms” [9]. With these experiments we could show that the effects of IR on the integrin β1 clustering are lipid raft independent. But our results also pose the question: who organizes whom? This is a well known question which is addressed for years in the filed of membrane research.

Our data indicates that this question has to be answered with “neither is responsible for the organization of the other”. While integrins and cholesterol rafts clearly colocalize under unstressed conditions, treatment with IR showed that lipid rafts cannot be made responsible for the clustered organization of integrins. In other words, cholesterol does not pattern integrins. On the other hand, the distribution of integrins turned out not to be responsible for the presence of cholesterol rafts, as disintegration of the former did not effect the latter. Hence, patterning processes behind cholesterol and integrins appear to be independent or at least lack strong mutual influence.

In conclusion, the generalized view of lipid rafts as an “organizing platform” is questioned by our data at least for integrins. In this respect our findings are also not in line with the general view that integrin-signalling stabilizes lipid rafts [30], as they remained stable in the absence of intact focal adhesions.

### Limitations

The present data do not provide a complete answer to the question on “Who organizes whom?”. Our results only imply that the generalized view of lipid rafts as organizing platforms has exceptions and needs further review.

## Additional file

**Additional file 1.** Additional information. Details of experimental procedure and Additional figures S1, S2 and S3.

## Abbreviations

ECM: extracellular matrix
SMD: single molecule detection
PM: plasma membrane
IR: ionizing radiation
CAM-RR: cell-adhesion-mediated-radio-resistance
FRAP: fluorescence recovery after photobleaching
PALM: photoactivated localization microscopy
CLSM: confocal laser scanning microscopy
FAK: focal adhesion kinase
