# Supplementary material for: Lipid-rafts remain stable even after ionizing radiation induced disintegration of β1 integrin containing focal adhesions
Source: BMC Res Notes. 2017 Dec 6;10:697. doi: 10.1186/s13104-017-3032-8 (PMC5717827; doi:10.1186/s13104-017-3032-8)
Supplement: Supplementary file 1 — Additional file 1. Additional information. Details of experimental procedure and Additional figures S1, S2 and S3. [file 13104_2017_3032_MOESM1_ESM.docx]

# Additional of:

# The plasma membrane located effects of ionizing radiation on β1 integrins contributing to radioresistance are lipid-raft independent

**Laura Babel^1,2^, Larissa Kruse^1^, Steven Bump^1^, Markus Langhans^1^, Tobias Meckel^1,2^**

## Experimental procedure

**Cell culture**

MEF cells (mouse embryonic fibroblasts, kindly provided by M. Loebrich, TU Darmstadt, Germany) were cultured in DMEM / Ham’s F-12 (1:1) (FG 4815, Biochrom, Berlin, Germany) supplemented with 10% FCS (F0804, Sigma-Aldrich, St. Louis, Missouri, USA) and 1% NEAA (K0293, Biochrom, Berlin, Germany). OV-MZ-6 cells (human ovarian serous cystadenocarcinoma, kindly provided by D. Loessner, Faculty of Health, Queensland University of Technology, Brisbane, Australia) were cultured in high glucose DMEM (12491-015, Thermo Fischer Scientific, Waltham, Ma, USA) supplemented with 10 mM HEPES buffer (15630080, Thermo Fischer Scientific, Waltham, Ma, USA), 0.27 mM L-asparagine (A4159, Sigma-Aldrich, St. Louis, Missouri, USA) and 10% FCS. All cells were cultured in a humidified chamber at 37 °C and 5% CO_2_. Both cell lines are frequently tested for mycroplasma contamination. Cell cultures were prepared on round coverslips (31224819, Karl Hecht GmbH, Sondheim, Germany, NO 1.5, Ø = 25 mm). The preparation of 3D cells was described in Babel et al. [[5]](https://paperpile.com/c/0qA8WC/rc5P). In brief, cells were cultured in 15 µl sized 1.5 mg/ml collagen I hydrogels (3 mg/ml rat-tail collagen I stock solution, A10483-01, Thermo Fischer Scientific, Waltham, Ma, USA) on APTS (3-Aminopropyl)triethoxysilane, 281778, Sigma-Aldrich, St. Louis, Missouri, USA) coated coverslips. 3D cells were incubated 4 to 5 days prior to use.

**FRAP**

FRAP experiments of OV-MZ-6 cells expressing CAAX-mCherry (pCDNA3.2) [[16]](https://paperpile.com/c/0qA8WC/VChlW) were performed with the LEICA SP5II (Leica Microsystems, Mannheim, Germany) by using a 562 LASER. Five pre-bleach images were recorded with a frame interval of 38 ms, followed by bleaching with a zoomed in region of interest (ROI) for 10 frames with an interval of 38 ms. 60 Post-bleach images were acquired with a time resolution of 38 ms, followed by 30 post-bleach images recorded with a time resolution of 2 s and finally 15 post-bleach images with a frame interval of 5 s were recorded, resulting in 105 post-bleach images for 137.28 s after bleaching. FRAP recovery curves were then normalized and fitted to a single- exponential function as published previously [[22]](https://paperpile.com/c/0qA8WC/mwEEC)**.**

**Radiation**

Cells were irradiated with an Isovolt 160 Titan E (GE Sensing & Inspection Technologies, Alzenau, Germany) x-ray source. Cells were irradiated with 15 Gy (voltage = 90 kV, current = 33.7 kV). Doses were delivered at a 30 cm source to probe distance with cell cultures placed on a 2 mm aluminum filtering plate with respect to the glass doubling factor [[23]](https://paperpile.com/c/0qA8WC/K7oyg).

**Immunostainings**

Integrin β1 (clone HMβ1-1, anti CD 29 Alexa Fluor 647 conjugate, 102214, Biozol Diagnostica, Eching, Germany) staining was performed according to our previous publication [[5]](https://paperpile.com/c/0qA8WC/rc5P).

**Lipid raft staining**

pET28/Dronpa-θ-D4 (stains cholesterol) plasmids were kindly provided by Dr. A. Miyawaki (RIKEN Brain Science Institute, Japan). Recombinant proteins were expressed in *E.coli* (BL 21) and purified using the Dynabeads His-tag isolation and pulldown kit (12320D, Thermo Fischer Scientific, Waltham, Ma, USA).

MEF cells were incubated in HBSS buffer (L2035, Thermo Fischer Scientific, Waltham, Ma, USA) containing 16 ng/ml Dronpa-θ-D4 or 60 ng/ml Dronpa-NT-Lys for 3 min on ice. Cells were washed quickly and were fixed with 4% PFA (0335.1, Carl Roth GmbH Karlsruhe, Germany) supplemented with 0.2% glutaraldehyde (23115, Serva Electrophoresis, Heidelberg, Germany) in PBS (P5493, Sigma-Aldrich, St. Louis, Missouri, USA, pH 6.9) for 1h at 4°C.

**Integrin - Lipid raft co-staining**

MEF cells were stained with the anti CD 29 Alexa Fluor 647 antibody (30 min / 37°C) in HBSS buffer followed by lipid raft staining and fixation.

**Cholesterol depletion**

MEF cells were incubated at 37°C for 30 min in HBSS buffer supplemented with 10 mM of MβCD (332615, Methyl-β-cyclodextrin, Sigma-Aldrich, St. Louis, Missouri, USA) followed by Dronpa-θ-D4 staining and fixation.

**Fixation control**

To see whether a storage of Dronpa-θ-D4 stained probes at 4°C influences the cholesterol clustering, one probe was measured directly after fixation and one 24 h after fixation.

**SMD measurements**

All SMD measurements were performed with a custom-built instrument. A detailed description of the setup was published previously [[5]](https://paperpile.com/c/0qA8WC/rc5P).

**Image acquisition and data analysis**

Editing of images was performed using Fiji (version: 1.51h) [[24]](https://paperpile.com/c/0qA8WC/uUuQq). To analyze the effects of ionizing radiation on β1 integrins and cholesterol rafts, single molecule signals were detected and filtered using the ThunderStorm plugin for Fiji [[25]](https://paperpile.com/c/0qA8WC/Fn7q5). For the add-on cluster analysis custom written software in MATLAB R2014b was used.

**Cluster analysis**

To analyze our single molecule data we used the Ripley’s K function. In brief, the result of this method is the so called H plot, where the degree of clustering (H(r) =L(r)-r) is plotted against a length scale. The maximum represents the most prominent cluster distribution. The height of the first local maximum (H(r)max) gives a measure of the degree of clustering and its position the radius of the most frequent clusters [[26–28]](https://paperpile.com/c/0qA8WC/ZS5yP+7CMNQ+70wLD). For statistical analysis confidence intervals of 68.27% were generated by simulating 100 random distributions with the same number of signals as a control data set. Pseudo colored heat maps were created similar to Williamson et al. [[29]](https://paperpile.com/c/0qA8WC/Uokaj), binary cluster maps were created based on the publication of Owen et al. [[30]](https://paperpile.com/c/0qA8WC/GAWdw). Binary cluster maps were used to determine the cluster density and the number of clusters per µm^2^. A detailed description of our cluster analysis can be found in Babel et al. [[5]](https://paperpile.com/c/0qA8WC/rc5P). To remove duplicates, molecules that convert to the same position were removed within the distance of the uncertainty of each dataset.

**Statistical analysis**

Statistical analysis was performed with GraphPad Prism 7. For multiple comparisons, significances were analyzed with a Kruskal-Wallis test with a Dunn’s multiple comparison post hoc test. For simple comparisons, significances were analyzed with a Mann-Whitney test. For all cases, p ≤ 0.05 was considered significant (*), p ≤ 0.01 very significant (**) and p ≤ 0.001 extremely significant (***). Also p ≤ 0.0001 (****) was noted. All presented box plots show as a central line the median, the top and bottom of each box are the first and third quartile, top and bottom line represent the maximum and minimum values. Outliers are colored in red.

## Additional figures

**S1: Time between fixation and probe measurement does not affect lipid raft organization.** Box plots of normalized medians of H(r) max (A) and r max (B) obtained from Ripley’s K function analyzed data sets. 2D cells were either measured directly after fixation (control) or after a storage time of 24 h (control + t). Statistical analysis was performed with a Mann-Whitney test, no significance was detected (ns).

**S2: Cholesterol depletion leads to a loss of cholesterol microdomains.** (A and B) Scatter plots of single molecule detections from cholesterol stained 2D cells. (A) Untreated control and (B) after cholesterol depletion, scale bar is 1 µm. (C) H-plots of the data from (A, blue) and (B, red) as well as an analysis of 100 random distributions of localizations containing the same number of signals as the control (confidence interval, gray). (D) Zoom-in of (C).

####

**S3: Detailed analysis of the effect of IR on various cholesterol raft parameters of 2D and 3D cultured cells.** Box plots or scatter plots of the change of clustering (H(r) max), cluster radius (r in nm), cluster density and the number of clusters per µm^2^ in response to a x-irradiation with 15 Gy. (A-D) Results for 2D cells. (E - H) Corresponding data of 3D cultured cells. Statistical analysis was performed with a Kruskal-Wallis test, no significance was detected.
